# Consumption of red meat and whole-grain bread in relation to biomarkers of obesity, inflammation, glucose metabolism and oxidative stress

**DOI:** 10.1007/s00394-012-0340-6

**Published:** 2012-03-18

**Authors:** Jukka Montonen, Heiner Boeing, Andreas Fritsche, Erwin Schleicher, Hans-Georg Joost, Matthias B. Schulze, Annika Steffen, Tobias Pischon

**Affiliations:** 1 Department of Epidemiology, German Institute of Human Nutrition Potsdam-Rehbruecke, Arthur-Scheunert-Allee 114-116, 14558 Nuthetal, Germany; 2 Department of Internal Medicine IV, University of Tuebingen, Tübingen, Germany; 3 Department of Pharmacology, German Institute of Human Nutrition Potsdam-Rehbruecke, Nuthetal, Germany; 4 Department of Molecular Epidemiology, German Institute of Human Nutrition Potsdam-Rehbruecke, Nuthetal, Germany; 5 Molecular Epidemiology Group, Max Delbrueck Center for Molecular Medicine (MDC), Berlin-Buch, Germany

**Keywords:** Red meat, Whole grain, Biomarkers, Glucose metabolism

## Abstract

**Purpose:**

To examine the association of red meat and whole-grain bread consumption with plasma levels of biomarkers related to glucose metabolism, oxidative stress, inflammation and obesity.

**Methods:**

Our cross-sectional study was based on 2,198 men and women who were selected as a sub-cohort for an investigation of biological predictors of diabetes and cardiovascular diseases from the European Prospective Investigation into Cancer and Nutrition-Potsdam study. Circulating levels of glycated hemoglobin, adiponectin, hs-CRP, gamma-glutamyltransferase, alanine-aminotransferase, fetuin-A, HDL-cholesterol and triglycerides were measured from random blood samples. Diet and lifestyle data were assessed by questionnaires, and anthropometric data were measured.

**Results:**

After multivariable adjustment, higher consumption of whole-grain bread was significantly (*P* trend <0.05) associated with lower levels of GGT, ALT and hs-CRP, whereas higher consumption of red meat was significantly associated with higher levels of GGT and hs-CRP when adjusted for potential confounding factors related to lifestyle and diet. Further adjustment for body mass index and waist circumference attenuated the association between red meat and hs-CRP (*P* = 0.19).

**Conclusions:**

The results of this study suggest that high consumption of whole-grain bread is related to lower levels of GGT, ALT and hs-CRP, whereas high consumption of red meat is associated with higher circulating levels of GGT and hs-CRP.

## Introduction

Results from epidemiological studies suggest that high consumption of red meat is associated with increased risk of cancers of colon and rectum, cardiovascular disease and type 2 diabetes [1–4], whereas high intake of whole grains is related to a reduced risk of these diseases [5–8]. The biological mechanisms explaining these associations are not completely understood. High consumption of red meat may increase insulin resistance, oxidative stress and inflammation [9–11], whereas high intake of whole grains may provide protection by improving insulin sensitivity, lowering insulin and glucose levels, and by inhibiting inflammation and oxidative stress [12]. Thus, oxidative stress measured as circulating levels of gamma-glutamyl transferase (GGT) was positively associated with red meat intake and inversely associated with whole-grain intake in a prospective analysis of the Coronary Artery Risk Development in Young Adults (CARDIA) Study [13]. In addition, cross-sectional studies have suggested an inverse association between whole-grain consumption and systemic inflammation [14–16], but recent trials on substitution of refined grain products with whole-grain wheat or oat have been inconclusive [17, 18]. A few recent cross-sectional analyses have shown positive associations between whole-grain consumption and circulating levels of adiponectin [19, 20] and negative associations with concentrations of triglycerides and LDL-cholesterol [21–25] but not for circulating HbA1c levels [16]. The existing literature relating consumptions of red meat with biomarkers of insulin resistance and inflammation is still scarce. In one recent analysis consumption of red meat was related with plasma CRP levels in ~500 women [26].

Since red meat and whole grains potentially have opposite effects on common pathways of chronic diseases, it is possible that their associations with disease risk may be modified by each other. However, to date no data exists on potential interactions between red meat and whole grains on circulating concentrations of biomarkers of inflammation, oxidative stress, disturbed glucose metabolism or dyslipidemia.

In the present study, we examined the association of red meat and whole-grain bread consumption with plasma levels of biomarkers related to glucose metabolism (glycated hemoglobin, HbA1c), insulin sensitivity (adiponectin), inflammation (C-reactive protein, CRP), oxidative stress (gamma-glutamyltransferase, GGT), hepatic fat accumulation (GGT; alanine-aminotransferase, ALT, and fetuin-A) and dyslipidemia (high density lipoprotein cholesterol, HDL-C, and triglycerides) and whether their associations with biomarker levels may be modified by each other.

## Subjects and methods

The European Prospective Investigation into Cancer and Nutrition (EPIC) Potsdam Study is part of the multicenter prospective cohort study EPIC, which was designed to investigate the association between nutrition, cancer and other chronic diseases [27, 28]. In Potsdam, Germany, 27,548 subjects (16,644 women aged 35–65 years and 10,904 men aged 40–65 years) from the general population were recruited between 1994 and 1998 [29]. The baseline examination included anthropometric measurements, a personal health interview including questions about prevalent diseases, a health questionnaire and blood sampling. Informed consent was obtained from participants; approval was given by the Ethical Committee of the State of Brandenburg, Germany.

The present study was based on a sub-cohort of 2, 500 individuals who were selected at random from all participants with blood sample available at baseline of the EPIC-Potsdam study population as part of a separate case-cohort studies on biomarkers and risk of type 2 diabetes and cardiovascular diseases [30, 31]. After exclusion of subjects with missing data for biomarkers or covariates or with implausible data on energy intake (<800 or >6,000 kcal/day), the analytical sample comprised 2,198 individuals (836 men and 1,362 women).

Usual food intake of the preceding year was assessed by a self-administered 148-item food frequency questionnaire. For each item, the frequency of consumption was asked in 10 categories, ranging from ‘never’ to ‘five times a day or more’. Pictures and portion sizes were given to estimate the quantities that were consumed. The consumed amount of each food item was calculated in grams per day. High reproducibility (*r* = 0.77) 6 months apart was observed for meat consumption and moderate reproducibility (*r* = 0.49) for bread consumption. In addition, high agreements with repeated 24 h dietary recalls were observed [32].

Plasma levels of ALT, GGT, HDL-C and hs-CRP were determined using the automatic ADVIA 1650 analyzer (Siemens Medical Solutions, Erlangen, Germany). Total adiponectin concentration was measured by ELISA (Linco Research, St. Charles, MI, USA) [33]. For determination of fetuin-A, an immunoturbidimetric method was used with specific polyclonal goat anti-human fetuin-A antibodies to human fetuin-A (BioVendor Laboratory Medicine, Modreci, Czech Republic) [34]. All assay procedures were performed as described by the manufacturer. Red blood cell levels of HbA1c were measured using HPLC according to the manufacturer’s instructions (Tosoh, Stuttgart, Germany). Observed HbA1c levels were slightly higher than what would be expected for this population. Using a prediction equation based on sex, age and waist circumference derived from the Tuebingen Family Study for type 2 diabetes [35], we observed a mean difference of 1.04 %-points between the observed and predicted levels. Because we speculate that this difference is due to the long-term storage condition, we subtracted the difference as a constant from the individual HbA1c levels.

Lifestyle characteristics, including regular physical exercise, smoking history and educational attainment as well as occupational activity level were assessed with a self-administered questionnaire and a computer-guided personal interview. Sports activity was defined as the mean time spent on leisure time physical activities during the summer and winter seasons (hours per week). Body weight, height and waist circumference were measured by trained staff according to standard procedures with participants wearing light underwear [36]. Body mass index was calculated by dividing weight (kg) by height (m) squared. The prevalence of diabetes mellitus at baseline was evaluated by a physician using information on self-reported medical diagnosis, medication records and dietary therapy.

The association between red meat and whole-grain bread consumptions with plasma biomarker levels was investigated using general linear models. Since the distributions of plasma biomarkers concentrations were right-skewed, log-transformed values were used in all analyses, and the strength of the associations was expressed as multivariable adjusted geometric means with 95 % confidence intervals of plasma biomarker levels in quintiles of red meat and whole-grain bread consumptions. We tested for effect modification by sex using cross-product terms. Since tests for the interaction were nonsignificant, we analyzed men and women combined. Analyses were adjusted for potential confounding factors including age, sex, education (in or no training, vocational training, technical school, or technical college or university degree), occupational activity (light, moderate or heavy), sports activity (0, 0.1–4, or 4 or more h/week), cycling (0, 0.1–2.4, 2.5–4.9 or 5 or more h/week), smoking (never, past or current ≤20 cigarettes/day or current >20 cigarettes/day), alcohol intake (0, 0.1–5, 5.1–10, 10.1–20, 20.1–40 or over 40 g/day) and energy intake. Potential confounding dietary variables in the models were chosen based on published dietary patterns related to consumptions of whole-grain bread and red meat in EPIC-Potsdam study population [37]. The dietary variables in whole-grain bread analyses were pasta, rice, pizza, vegetarian dishes, muesli, refined grain bread, wine and spirits and in red meat analyses the corresponding dietary variables were cabbages, cooked vegetables, legumes, cooked potatoes, mushrooms, sauce and poultry. A model including body mass index and waist circumference was also used to describe in which extent the associations observed are potentially mediated by obesity. Tests for a linear trend across quintiles were performed by modeling the median value of dietary variables in each quintile as a continuous variable. All statistical analyses were performed with SAS release 9.2 (SAS Institute, Cary, NC, USA). All statistical tests were two-sided, and *P* values <0.05 were considered statistically significant.

## Results

Subjects in the highest quintile of whole-grain bread consumption were less likely to be men, and smokers and they reported more biking and sports activity than persons in the lowest quintile of whole-grain bread (Table 1). They also more often had university degree education. Of the food components of the dietary pattern related to whole-grain bread consumption, pizza, muesli, vegetarian dishes, wine were directly and bread other than whole-grain bread and spirits were inversely associated with whole-grain bread consumption. Of the food components of dietary pattern related to red meat consumption, whole-grain bread consumption was directly associated with cooked vegetables and inversely with cooked potatoes. Subjects with higher red meat consumption were more likely to be men, smokers and less active, and they reported higher alcohol and coffee consumptions and they more often had prevalent diabetes than subjects with lower meat consumption. Body mass index and waist circumference showed increasing trends across the quintiles of red meat consumption. The Pearson correlation coefficient for whole-grain bread consumption with red meat intake was −0.12.

**Table 1:** Characteristics by quintiles of red meat and whole-grain consumption

	Quintiles of whole-grain bread			*P* trend^a^	Quintiles of red meat			*P* trend^a^
	Quintile 1 0–4.75 g/day	Quintile 3 15.9–38.1 g/day	Quintile 5 79.8–362 g/day		Quintile 1 0–20.2 g/day	Quintile 3 31.6–42.0 g/day	Quintile 5 59.6–187 g/day	
Age, years^b^	51.1	49.4	50.7	0.60	49.5	51.2	49.9	0.93
Sex, % men^c^	47.2	36.8	33.3	<0.01	31.8	38.7	45.5	<0.01
Energy intake, kJ/day^d^	8,501	8,649	8,870	<0.01	7,878	8,294	9,643	<0.01
Whole-grain bread, servings (50 g)/day	0.02	0.52	2.68	<0.01	1.17	0.90	0.69	<0.01
Red meat, servings (150 g/day)	0.31	0.28	0.24	< 0.01	0.08	0.25	0.58	<0.01
Sport activity, h/week	0.75	0.96	1.30	<0.01	1.30	1.00	0.85	<0.01
Biking, h/week	1.44	1.78	2.27	<0.01	2.19	2.02	1.47	<0.01
Body mass index, kg/m^2^	26.1	26.3	26.0	0.51	25.6	26.3	26.7	<0.01
Waist circumference, cm	86.3	86.0	85.4	0.17	84.6	85.5	87.5	<0.01
Prevalent diabetes, %	4.26	5.21	4.50	0.96	1.81	4.23	8.23	<0.01
Prevalent myocardial infarction, %	2.58	1.40	1.53	0.29	2.20	2.01	2.88	0.45
Prevalent stroke, %	2.02	1.01	0.94	0.26	1.78	1.11	0.52	0.15
Heavy work strain, %	8.86	7.16	5.82	0.13	8.32	7.35	6.39	0.31
University degree education, %	27.2	42.3	40.6	<0.01	35.8	36.7	38.6	0.25
Current- or ex smoker, %	60.3	53.9	51.3	0.02	51.3	49.5	60.4	<0.01
Coffee, cups/day	2.96	2.83	2.61	0.02	2.58	2.72	3.12	<0.01
Alcohol consumption, g/day	14.0	14.4	12.4	0.02	11.9	13.3	15.9	<0.01
Components of dietary pattern related to whole-grain bread intake, g/day								
Pizza	5.57	8.01	7.78	<0.01	7.10	7.38	6.91	0.89
Pasta and rice	14.7	16.9	16.5	0.12	14.8	16.2	18.6	<0.01
Muesli	4.24	5.40	8.78	<0.01	9.93	3.78	1.49	<0.01
Vegetarian dishes	0.50	1.39	2.80	<0.01	3.12	1.16	0.76	<0.01
Wine	37.4	56.5	66.1	<0.01	50.5	46.6	55.9	0.52
Spirits	4.45	2.47	1.68	<0.01	1.76	2.53	3.80	<0.01
Bread other than whole-grain bread	161	135	70.1	<0.01	118	129	122	0.57
Components of dietary pattern related to red meat intake, g/day								
Sauce^e^	12.3	13.1	11.6	0.26	8.24	11.6	18.4	<0.01
Cooked vegetables	28.2	28.7	31.2	0.01	25.0	28.4	37.2	<0.01
Cooked potatoes	89.9	77.1	79.3	<0.01	61.6	81.5	109	<0.01
Cabbage family	14.5	14.5	14.8	0.60	10.3	14.7	18.9	<0.01
Poultry	12.2	13.1	12.6	0.87	7.39	12.2	20.6	<0.01
Mushrooms	2.29	2.00	2.28	0.19	1.63	1.79	2.92	<0.01
Legumes	22.5	22.3	22.4	0.59	17.7	22.0	27.6	<0.01

Adjusted for age, sex and energy intake with an exception when as outcome variable

^a^Calculated by using the median values within quintiles as a continuous variable in linear model adjusting for age, sex and energy intake

^b^Not adjusted for age

^c^Not adjusted for sex

^d^Not adjusted for energy intake

^e^Ketchup, brown and white sauce, salad dressing, sauce for vegetables

In our multivariable model, a modest but significant inverse association of whole-grain bread consumption was observed for circulating levels of GGT, ALT and hs-CRP (*P* trend across quintile medians (*P*) <0.05), but not for adiponectin, HDL-cholesterol, triglycerides or HbA1c (Table 2). Results remained virtually unchanged after additional adjustment for body mass index and waist circumference (data not shown). For example, geometric mean serum GGT levels across the quintiles of whole-grain bread consumption were 24.3 (CI 22.1–26.8), 22.2 (CI 20.1–24.5), 21.1 (CI 19.1–23.2), 22.5 (CI 20.4–24.8) and 19.8 (CI 17.9–21.9), with *P* = 0.003. Further adjustment for cooked potatoes and cooked vegetables did not notably alter the results (data not shown), neither did exclusion of subjects with history of diabetes, myocardial infarction or cerebrovascular stroke (data not shown).

**Table 2:** Association of whole-grain bread and red meat consumption with biomarker levels

	Quintiles of whole-grain bread intake					*P* trend^a^
	1 (0–4.75 g/day)	2 (4.77–15.9 g/day)	3 (15.9–38.0 g/day)	4 (38.1–79.5 g/day)	5 (79.8–362 g/day)	
Adiponectin, ng/L^b^	7.03 (6.61–7.46)	6.83 (6.41–7.27)	7.09 (6.66–7.54)	6.78 (6.36–7.21)	6.93 (6.50–7.40)	0.76
GGT, U/L	24.7 (22.3–27.2)	22.5 (20.3–25.0)	21.5 (19.4–23.8)	22.9 (20.7–25.4)	20.0 (18.0–22.3)	0.004
ALT, U/L	22.5 (21.2–23.9)	21.4 (20.1–22.8)	20.7 (19.5–22.0)	21.6 (20.3–23.0)	20.2 (18.9–21.6)	0.02
hs-CRP, mg/L	0.77 (0.65–0.92)	0.86 (0.71–1.03)	0.91 (0.76–1.09)	0.79 (0.66–0.95)	0.68 (0.56–0.82)	0.02
HDL-cholesterol, mmol/L	1.31 (1.27–1.35)	1.29 (1.25–1.34)	1.29 (1.24–1.34)	1.29 (1.24–1.33)	1.28 (1.23–1.33)	0.33
Triglycerides, mmol/L	1.25 (1.16–1.34)	1.29 (1.19–1.39)	1.24 (1.15–1.34)	1.30 (1.21–1.40)	1.23 (1.14–1.33)	0.56
HbA1c, %	5.54 (5.44–5.64)	5.54 (5.44–5.64)	5.58 (5.48–5.69)	5.65 (5.54–5.75)	5.55 (5.45–5.66)	0.72
Fetuin, μg/mL	247 (239–256)	244 (236–253)	236 (229–245)	243 (234–251)	239 (231–248)	0.26

	Quintiles of red meat intake					
	1 (0–20.2 g/day)	2 (20.3–31.5 g/day)	3 (31.6–42.0 g/day)	4 (42.1–59.5 g/day)	5 (59.6–187 g/day)	
Adiponectin, ng/L	7.09 (6.66–7.55)	7.02 (6.59–7.48)	7.11 (6.68–7.57)	6.80 (6.39–7.24)	6.99 (6.55–7.47)	0.56
GGT, U/L	21.9 (19.7–24.3)	21.5 (19.3–23.8)	21.6 (19.5–23.9)	22.2 (20.1–24.6)	25.4 (22.8–28.3)	0.007
ALT, U/L	21.1 (19.9–22.5)	21.2 (19.9–22.6)	20.8 (19.6–22.2)	21.4 (20.1–22.7)	22.4 (21.0–23.9)	0.10
hs-CRP, mg/L	0.73 (0.61–0.88)	0.74 (0.61–0.89)	0.78 (0.65–0.94)	0.87 (0.72–1.04)	0.88 (0.73–1.07)	0.05
HDL-cholesterol, mmol/L	1.31 (1.26–1.36)	1.28 (1.24–1.33)	1.29 (1.25–1.34)	1.27 (1.23–1.32)	1.32 (1.27–1.37)	0.68
Triglycerides, mmol/L	1.26 (1.17–1.36)	1.20 (1.11–1.29)	1.23 (1.14–1.33)	1.27 (1.18–1.37)	1.31 (1.21–1.41)	0.15
HbA1c, %	5.52 (5.41–5.62)	5.57 (5.47–5.68)	5.61 (5.50–5.71)	5.61 (5.51–5.72)	5.56 (5.45–5.67)	0.52
Fetuin, μg/mL	243 (235–252)	246 (238–255)	241 (233–249)	244 (236–253)	237 (228–245)	0.14

^a^Calculated by using the median values within quintiles as a continuous variable in linear model

^b^Geometric mean values with 95 % confidence interval, adjusted for age, sex, education, sport activity (0, 0.1–4, or >4 h/week), occupational activity (light, moderate, heavy), smoking (never, past or current <20, current 20 or more cigarettes/day), alcohol intake (0, 0.1–5, 5.1–10, 10.1–20, 20.1–40 or >40 g/day) and dietary variables (continuous): energy intake, consumptions of red meat, coffee and food items aggregating into same dietary pattern with whole-grain bread (pasta and rice, pizza, vegetarian dishes, muesli, other bread than whole-grain bread, wine and spirits) and food items aggregating into same dietary pattern with red meat (cabbages, cooked vegetables, legumes, cooked potatoes, mushrooms, sauce and poultry), respectively

A significant (*P* trend <0.05) positive association between red meat consumption and plasma levels of GGT and hs-CRP was observed (Table 2). After further adjustment for BMI and waist circumference, only the association with GGT remained significant. In that model, the adjusted geometric mean values of GGT (U/L) across quintiles of red meat consumption were 22.0 (CI 19.4–24.3), 21.2 (CI 19.2–23.5), 21.5 (CI 19.5–23.7), 21.7 (CI 19.7–24.0) and 24.6 (CI 22.2–27.3), with *P* = 0.03 (data not shown). We also included protein intake in the final model and observed no notable change in the strength of the association, although the *P* value for trend across quintiles was no longer significant at the 5 %-level (*P* = 0.08). Exclusion of subjects with history of diabetes, myocardial infarction or cerebrovascular stroke did not notably alter the result (data not shown).

**Fig. 1:**
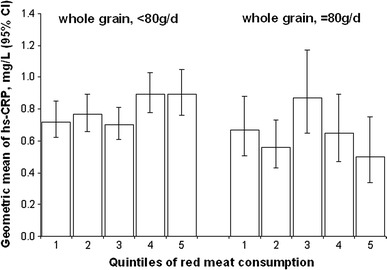
Geometric means (95 % CI) of plasma hs-CRP levels across quintiles of red meat according to categories of whole-grain bread consumption [adjusted for age, sex, education (sport activity (0, 0.1–4, or >4 h/week), occupational activity (light, moderate, heavy), smoking (never, past or current <20, current 20 or more cigarettes/day), alcohol intake (0, 0.1–5, 5.1–10, 10.1–20, 20.1–40 or >40 g/day) and dietary variables (continuous): energy intake, consumptions of coffee and food items aggregating into same dietary pattern with red meat such as cabbages, cooked vegetables, legumes, cooked potatoes, mushrooms, sauce and poultry and food items aggregating into same dietary pattern with whole-grain bread, such as pasta and rice, pizza, vegetarian dishes, muesli, other bread than whole-grain bread, wine and spirits. *P* for interaction = 0.02]

To explore the joint association of red meat and whole-grain bread with plasma GGT and hs-CRP values, we cross-classified individuals on the basis of quintiles of consumption of red meat and whole-grain bread. In this analysis, the positive association between red meat consumption and plasma levels of hs-CRP and GGT was less pronounced in participants in the highest quintile of whole-grain bread consumption in comparison with individuals in the lowest quintile of whole-grain consumption (data not shown). We then combined the lower 4 quintiles of whole-grain bread consumption and compared the associations between red meat intake and circulating biomarker levels in persons with whole-grain bread consumption in the highest quintile and in persons in the lower quintiles. While no significant interaction was observed for GGT (*P* = 0.48), a significant interaction was found for CRP (*P* = 0.02). Figure 1 therefore displays the geometric mean values (mg/L) of hs-CRP across quintiles of red meat intake in categories of whole-grain intake (highest quintile vs. lower quintiles). Generally, lower hs-CRP levels were observed among subjects in the highest quintile of whole-grain bread (with *P* = 0.44) than among subjects in the lower quintiles (with *P* = 0.049).

## Discussion

In the present analyses, we found that high consumption of whole-grain bread was significantly associated with modestly lower levels of GGT, ALT and hs-CRP, whereas high consumption of red meat was significantly associated with higher levels of GGT and hs-CRP after adjustment for potential confounding factors related to lifestyle and diet. After further adjustment for body mass index and waist circumference, the association between red meat consumption and hs-CRP was no longer statistically significant, suggesting that the degree of adiposity may explain this association and obesity may thus be considered as a potential intermediate factor. The results of the present study also suggest that the relation of red meat consumption with higher hs-CRP levels may be modified by higher whole-grain bread consumption or factors closely related to it.

The observed inverse association between whole-grain bread consumption and plasma CRP levels is in line with a cross-sectional analysis among 902 diabetic women in the Nurses’ Health Study, which suggested that consumption of whole grains and cereal fiber was associated with lower inflammatory markers such as CRP and soluble TNF receptor 2 [14]. Another study in a multi-ethnic sample of 5,496 men and women suggested an inverse association between whole-grain consumption and CRP levels [15]. However, in an analysis based on a subset of men from the Health Professionals Follow-Up Study and women from the Nurses’ Health Study II, the significant association between whole-grain intake and serum CRP disappeared after adjustment for lifestyle factors, including alcohol intake, smoking, BMI, physical activity and hypercholesterolemia [16]. Furthermore, recent trials on the effect of substitution of refined grain products with whole-grain wheat or oat products have been inconclusive [17, 18]. In recent comprehensive intervention trials among 316 overweight participants [18] and among 206 participants with normal weight [17], neither three servings of whole-grain foods per day for 12 weeks nor 120 g/day for 16 weeks significantly affected plasma CRP levels. In both interventions, the authors discussed that diets of high whole-grain consumers in these intervention studies were different from that of high whole-grain consumers in observational studies, and whole-grain consumption may be a marker of an otherwise healthier diet. However, in the present study, the association was observable even after comprehensive adjustment for dietary factors. The null findings in intervention studies suggest that the observation may still be confounded.

Of the biomarkers of dyslipidemia, glucose metabolism and insulin sensitivity consumption of whole-grain foods were related to lower LDL-cholesterol but not to HDL-cholesterol or triglyceride or HbA1c concentrations after adjustment for lifestyle and dietary factors in Framingham Offspring Cohort Study [15, 16]. Consumption of whole-grain foods was also associated with lower triglyceride concentrations in 827 men and women from the Tehran Lipid and Glucose Study [25]. Among men and women with diabetes from the Health Professional Follow-up Study and Nurses’ Health Study cereal fiber intake was associated with higher plasma adiponectin levels [19, 20]. In the present study, no association was observed between consumption of whole-grain bread and HbA1c or plasma values of HDL, triglycerides or adiponectin.

In the present study, the positive association between red meat intake and plasma hs-CRP levels did not remain significant after adjustment for BMI and waist circumference. Since the adipose tissue, particularly from visceral fat depots, is capable of producing pro-inflammatory cytokines that induce hepatic production of CRP and other acute phase proteins, it is plausible that the association observed is mediated by obesity. Inflammation as well as oxidative stress has been suggested to be involved in the pathogenesis of diabetes, cardiovascular diseases and other obesity-related diseases [38, 39]. However, in a recent cross-sectional analysis among 482 Iranian women, the association between red meat consumption and CRP persisted adjustment for BMI [26]. The liver enzymes GGT and ALT have been proposed as markers of accumulation of hepatic fat, which is related to increased gluconeogenesis and decreased glycogen storage in the liver [40]. In the present study, a significant association observed between red meat consumption and GGT persisted after adjustment for measures of obesity, and no association was observed between ALT and red meat consumption. Since GGT is also a potential nonspecific marker of oxidative stress [41–44], it is plausible that increased oxidative stress plays an important role in the association between red meat consumption and development of chronic diseases. Also in line with these results, meat consumption was linearly associated with increased serum GGT levels measured after 10 years in CARDIA study [13] after adjustment for BMI and other lifestyle and dietary factors. Besides obesity, the high consumption of red meat may increase inflammation and oxidative stress because of the high intake of saturated fat, protein, heme-iron, nitrates and nitrite, heterocyclic amines and polycyclic aromatic hydrocarbons, or glycation end products that are usually contained in prepared red meat [45–51]. Nevertheless, in an intervention study, a daily intake of ~200 g red meat for 8 weeks among 60 healthy subjects did not elevate plasma levels of GGT or CRP compared to a control group [52].

Several prospective studies have suggested that high red meat consumption is associated with a modestly increased risk of colorectal, colon and rectal cancers, cardiovascular disease and type 2 diabetes [1–4], whereas high whole-grain intake has been related to reduced risks of colorectal, colon and rectal cancers, cardiovascular diseases and diabetes [5–8]. The association of type 2 diabetes risk with higher consumption of red meat and lower consumption of whole-grain bread has been observed in the study population of the present study [53]. Since circulating levels of GGT and hs-CRP have been related in development of diabetes and cardiovascular disease in several prospective studies [54–57], it seems possible that part of the association between red meat and disease risk may be modified by whole-grain intake. The finding in the present study suggests that the positive association between red meat consumption and circulating levels of hs-CRP depends on consumption of whole-grain bread. However, this finding needs to be verified.

The present study was conducted in a cross-sectional design, which complicates the drawing of causal inferences. In the present study, we had data available from a single blood drawing only, which might have introduced random measurement error in determining biomarkers. However, most of the markers presented here have been shown to have a suitable reliability for epidemiological studies [58, 59]. The dietary data were collected using a food frequency questionnaire (FFQ), which is a common tool in nutritional epidemiology to rank the participants according to their food consumption. FFQs tend to overestimate the amount of consumed foods when compared to dietary recalls and food diaries, therefore the absolute amount of food intake must be interpreted cautiously [60]. However, fair to good reliability and agreement with 24 h recall data of assessment of red meat and bread consumption have been observed in this study population [32]. In addition, we based our analysis of whole-grain bread and red meat and their interaction with biomarker levels on quintile distribution, therefore, nondifferential misclassification of food intake should not have affected the results.

In summary, the results of this study suggest that high red meat consumption is associated with modestly higher concentrations of plasma GGT and hs-CRP, whereas high whole-grain bread consumption is related to modestly lower concentrations of GGT, ALT and hs-CRP. The association observed between red meat and hs-CRP is dependent on obesity. This study also suggests that the cross-sectional association of red meat consumption with increased levels of hs-CRP could be modified by high whole-grain bread consumption. Although the strengths of these associations were modest, our observations highlight the hypothesis that dietary factors may modulate these biomarkers, which may be potential mediators related to risk of diabetes and CVD.
